# Digital Pathology Standards: A Response to WG-26

**DOI:** 10.1016/j.jpi.2025.100510

**Published:** 2025-08-22

**Authors:** Peter Gershkovich

**Affiliations:** Pathology Informatics & Cancer Data Science, Yale Medical School, Pathology Department, New Haven, CT, USA

## Dear Editors,

Thank you to WG-26 for their response and the opportunity for continued dialogue. One of the key motivations of my research is an absence of critical re-evaluations of DICOM in light of ongoing technological transformation—both theoretical and practical. This is a necessary discourse that I hope will grow to include a variety of perspectives.

Here is how I see the situation on the ground at my particular institution: Yale School of Medicine. My group and I have demonstrated the feasibility of rapid, effective integration of digital slides with custom laboratory information systems (LIS). This has enabled functionalities that are otherwise difficult to achieve, such as side-by-side display of batch positive controls with corresponding immunostains, implementation of a scalable deidentification pipeline, and streamlined slide collection workflows for validation studies and tumor boards. These solutions were delivered within weeks and they run on existing modular technologies with marginal cloud costs for deployment. By contrast, our review of DICOM-based approaches revealed a level of complexity and cost that often appeared unnecessary for pathology-specific use cases. In this context, I felt it was timely and important to raise these issues within the field. And having shared my thoughts with colleagues, I was encouraged to do so by their support and endorsement.

After reviewing the WG-26 response, I believe that my fundamental argument—that digital pathology represents a unique inflection point where we must critically evaluate inherited standards—remains unchallenged. The success stories cited in their response to my article rely on institutional resources not available to most pathology departments, which highlights a disparity in funding, and ultimately illustrates isolated feasibility rather than generalizable solutions.^1^ Michigan Medicine's implementation, a remarkable achievement presented in detail at the 2025 Pathology Informatics Summit in May, heavily leveraged generously donated surplus radiology infrastructure and unusually large departmental funds. Most pathology groups, it seems, face a different reality—a lack of funding, little institutional desire to invest in Digital Pathology, outdated PACS systems unable to support pathology, and vintage non-cooperative LISs allowing for minimal integration.

Still more significantly, the recent Connectathon cited by WG-26 explicitly defined performance, security, and image-quality metrics out of scope—precisely the operational parameters that matter in routine digital diagnostics. As I argued in the article, true interoperability must be functional and comprehensive; it must go well beyond simple file transfer. My phrase “elusive interoperability” underscores how the term is too often reduced to bare-bones exchange rather than dependable, end-to-end capability. When the organizing committee states, “Connectathon is about interoperability, not performance or quality; evaluating those is outside our mandate,” the disconnect between standards development and clinical reality becomes evident.^2^ That gulf is widened further by treating the Anatomic Pathology LIS as a mere metadata provider instead of the central orchestrator that drives the entire pathology workflow from specimen receipt through final diagnosis and reporting. Yes, DICOM can be used in digital pathology; the open question—and one that deserves rigorous scrutiny—is whether it is the optimal choice.

Another core argument—that embedding mutable clinical metadata inside immutable image containers introduces greater risk than benefit—was not substantively addressed by the WG-26 critique. Embedding mutable, sensitive clinical metadata within image files is fundamentally at odds with National Institute of Standards and Technology (NIST's) zero trust architecture, which requires minimizing implicit trust and strictly controlling access to protected data.^3^ This approach increases the risk of unauthorized disclosure and undermines cryptographic guarantees of data integrity, as any change to embedded metadata disrupts verifiable provenance and auditability. Further, as my article demonstrates, treating embedded metadata as authoritative without having access to source systems creates dangerous assumptions. Mutable metadata fundamentally conflicts with cryptographic integrity. Meanwhile, the disaster scenario presented in the WG-26 response, where all external systems fail but DICOM viewers remain functional is strategically speculative. Pathology diagnosis inherently requires access to broader clinical resources beyond individual images to follow with the pathological diagnosis and treatment plan.

## Beyond the DICOM paradigm

Pathology workflows are inherently specimen-centric and patient-centric—not image-centric. LIS, and their integration with Electronic Health Records, have evolved into highly complex ecosystems that rely on rich, extensible data models—typically implemented through databases and exchanged via HL7-based messaging standards. Digitized slides are data assets, and like other digital elements in pathology, they require data management systems—not just image viewers. From the LIS perspective, whole-slide images are simply one of many digital components associated with a case. If DICOM cannot represent the complete clinical picture, it should not attempt to embed mutable fragments of it—particularly when doing so creates the illusion of integrity without source-system verification. For AI tools to be clinically useful, they must interface with all relevant sources of patient and diagnostic data—not just the images alone.

I advocate for a modular, pathology-centered architecture that leverages modern technologies from the broader informatics ecosystem. Emerging and widely adopted standards such as HL7 FHIR and RESTful APIs in general exemplify how versioned, well-documented, lightweight, and developer-friendly interfaces can support interoperability while fostering innovation. Security and operational integrity are best achieved not by rigid encapsulation but through layered controls informed by frameworks such as those developed by NIST—now a de facto authority on cybersecurity architecture across industries.

Digital pathology stands at a pivotal moment. Emerging reliance on commoditized PACS frameworks—systems that often compete on acquisition price but entail significant downstream costs, limited adaptability, and constrained innovation—risks subordinating pathology to the priorities of radiology-centric platforms. In contrast, a commitment to open, flexible standards and purpose-built tools can re-establish pathology's role as a “microcosm of medicine”—driving diagnostic innovation, enabling precision workflows, and directly improving patient care.^4^

This conversation must extend beyond WG-26 and engage the broader pathology, informatics, engineering, cybersecurity, and regulatory communities. A collaborative, transparent, and evidence-based dialogue is essential to ensure that the standards guiding digital pathology truly serve patient care and diagnostic excellence.

## Declaration of competing interest

The authors declare the following financial interests/personal relationships which may be considered as potential competing interests:

Peter Gershkovich reports a relationship with Applikate Technologies, Inc. that includes: consulting or advisory. If there are other authors, they declare that they have no known competing financial interests or personal relationships that could have appeared to influence the work reported in this paper.
